# Traumatic Brain Injury and Hypopituitarism

**DOI:** 10.1100/tsw.2005.100

**Published:** 2005-09-15

**Authors:** Gianluca Aimaretti, Ezio Ghigo

**Affiliations:** Division of Endocrinology and Metabolism, Department of Internal Medicine, University of Turin, Corso Dogliotti 14, 10126,, Turin, Italy

**Keywords:** traumatic brain injury (TBI), hypopituitarism, pituitary deficits

## Abstract

Results of recent and ongoing studies have made it clear that brain injuries like Traumatic Brian Injury (TBI) pose substantial risk to pituitary function, perhaps even greater risk than previously believed. Patients with TBI should be screened both prospectively and retrospectively for isolated, multiple and even total pituitary deficits. It is well known that, patients with “classical” hypopituitarism (due to primary hypothalamic-pituitary pathologies) do benefit from hormonal replacement therapy. It has been suggested that patients with TBI-induced hypopituitarism may benefit with appropriate hormonal replacement receiving replacement therapy such as anti-diuretic hormone (ADH), glucocorticoid and thyroid hormones when needed. Gonadal and recombinant human Growth Hormone (rhGH) replacement therapy should also be introduced if there are deficiencies demonstrated and even reconfirmed in a second step. The signs and symptoms of post-TBI hypopituitarism may be masked by what has been assumed to be merely the post-traumatic syndrome. By increasing awareness among physicians of the risks of brain injuries–induced endocrinopathies and the need for appropriate endocrinological testing, it may be possible to improve the quality of life and enhance the rehabilitation prospects for these patients. In most instances, these patients are first seen and treated by trauma surgeons and neurosurgeons, and subsequently by rehabilitation physicians; they must be knowledgeable about the risks of hypopituitarism so that they can determine which patients are candidates for screening for hypopituitarism. In addition, endocrinologists and internists must be educated about TBI-induced hypopituitarism and encouraged to actively share their expertise with other physicians.

## INTRODUCTION

The first report of traumatic brain injury (TBI)–induced hypopituitarism was published in 1918[1]. Subsequent reviews and case reports identified additional instances of hypopituitarism following head injury[2,3,4]. One particular study that alerted endocrinologists of the relationship between TBI and hypopituitarism was published in 2000 by Salvatore Benvenga and colleagues and included 367 patients with TBI-induced hypopituitarism together with new data from 15 patients from their own center. They found that in most cases (71%), hypopituitarism was diagnosed within 1 year of injury, though occasionally diagnoses were not made for more than 20 years after the injury. Indeed, there are reports of patients with total, multiple, or isolated hypopituitarism whose clinical history revealed TBI many years before the diagnosis, but the patients themselves did not recall the brain injury without prompting or the assistance of a friend or family member[5].

## EPIDEMIOLOGY OF TRAUMATIC BRAIN INJURY

A wide range of scientific studies have attempted to evaluate the epidemiology of TBI. Because of differences in study inclusion criteria, a valid indicator of the incidence of TBI could be the rate of hospitalizations for TBI. These ranged from 91 per 100,000 patients in Cantabria, Spain[6] to upwards of 300 per 100,000 hospital admissions in Italy (Emilia Romagna and Trentino), South Africa (Johannesburg), southern Australia, and Scotland[7,8,9,10]. According to Thurman and colleagues, there were 230,000 hospital admissions annually in the U.S. with an incidence of nearly 100 per 100,000[11]. TBI is the leading cause of death and disability in the U.S. alone. As of 1998, 1.5 to 2 million TBI patients were reported annually and more than 50,000 resulted in death (25,000 of which were the direct result of the trauma itself). Each year, 70,000 to 90,000 people develop a significant loss of function. The National Institutes of Health Consensus Development Panel on Rehabilitation of Persons with TBI estimated that 2.5 to 6.5 million people in the U.S. are living with TBI[12].

## TRAUMATIC BRAIN INJURY AND HYPOPITUITARISM: REVIEW OF RECENT SCIENTIFIC LITERATURE

In 1986, Edwards and Clark reviewed the topic of post-traumatic hypopituitarism and found that the typical patient was a young adult male who had experienced a severe form of head trauma and who presented with endocrine abnormalities within months to years after the accident. These patients could have reported temporary or permanent diabetes insipidus or other clinical symptoms such as weight loss, fatigue, faintness, loss of libido, and impotence. The ensuing hypopituitarism was usually ascribed to depression or the “postconcussion syndrome” with inappropriate treatment and rehabilitation often recommended. Following these observations, the authors recommended that patients with major head injury (defined by post-traumatic amnesia greater than 24 h) and, in particular, those with fractures of the base of the skull or diabetes insipidus, be closely monitored for signs and symptoms of endocrine dysfunction and appropriate dynamic pituitary-function tests performed[4].

In 2000 and 2001, Kelly et al. and Lieberman et al. found a high prevalence of anterior pituitary dysfunction in patients with a previous history of moderate and severe TBI[13,14]. The frequencies of growth hormone deficiency (15%) and low cortisol levels (46%) were striking and may have had important implications for the patients’ health, sense of well being, and rehabilitation potential[13]. Kelly and colleagues emphasized also that patients with subarachnoid hemorrhage (SAH) may also be at high risk for ensuing hypopituitarism[13]. This clinical picture was more recently confirmed by additional studies in both TBI and SAH[15,16,17,18,19,20,21,22]. The percentage of patients with hypopituitarism after TBI or SAH varied between 20 and 80%. The studies were primarily retrospective; some tested patients who had experienced brain injury more than a year ago, others tested patients early after TBI or SAH[13,14,16,17,18,19,20,22,23]. All authors emphasized the clinical relevance of brain injury–induced anterior pituitary dysfunction and hypothesized that appropriate hormonal replacement may provide notable improvement of symptoms related to the post-traumatic syndrome as well as enhance the recovery of these patients.

The high risk for TBI-induced hypopituitarism and, particularly, for anterior pituitary dysfunction, was also clear in our studies (performed under the auspices of the Italian Society of Endocrinology) where we evaluated pituitary function 3 and 12 months after TBI (n = 100) or SAH (n = 40)[21,22]. The prospective, multicenter study assessed pituitary function during the course of 1 year after brain injury, specifically investigating whether pituitary deficiencies and normal pituitary function recorded at 3 months would improve or worsen, respectively, at 12 months after the brain injury. Results confirmed the high and similar risk for anterior pituitary dysfunction in both TBI and SAH patients, demonstrating that early diagnosis of total hypopituitarism is always confirmed at the long term after the brain injury while partial impairment of pituitary function (isolated and even multiple deficits) recorded just after brain injures may improve over time. Conversely, normal pituitary function at short term, although rarely, becomes impaired later on. Thus, brain-injured patients must always undergo neuroendocrine follow-up over time in order to monitoring pituitary function and eventually providing appropriate hormonal replacement.

## POSSIBLE PATHOGENESIS OF TBI-INDUCED HYPOPITUITARISM

The pathogenesis of brain injury–induced anterior pituitary dysfunction is not fully understood. Older studies in patients with fatal TBI demonstrated that various degrees of pituitary hemorrhagic infarction were present in more than 70% of patients[24,25], while hypothalamic microhemorrhages were present in at least 40% of patients[26]. Typically, pituitary stalk lesions are not considered a major cause of brain injury–induced hypopituitarism[25]. Furthermore, it is noteworthy that hypopituitarism secondary to brain injury can occur regardless of the severity of the neurological status after the brain injury[13,20,21,22]. Identification of lesions in the hypothalamic-pituitary region can account for the high incidence of hypopituitarism. However, these kinds of lesions also raise the possibility that anterior pituitary dysfunction identified soon after brain injury could either be progressive or, alternatively, it could be transient and pituitary function could recover over time.

## CONCLUSIONS

1. Brain injuries like TBI and SAH pose high risk to pituitary function.
2. Patients with TBI should be screened both prospectively and retrospectively for isolated, multiple, and total pituitary deficits.
3. Patients with TBI-induced hypopituitarism may benefit with appropriate hormonal replacement receiving replacement therapy when needed.
4. The signs and symptoms of TBI-induced hypopituitarism may be masked by what has been assumed to be the classical post-traumatic syndrome.
5. It is indicated to increase awareness among physicians of the risks of brain injuries–induced endocrinopathies and the need for appropriate endocrinological testing and substitutive therapies. These patients are first seen and treated by trauma surgeons and neurosurgeons, and subsequently by rehabilitation physicians who must be knowledgeable about the risks of hypopituitarism, in order so that they can determine which patients are candidates for screening for hypopituitarism. Endocrinologists and internists must be educated about TBI-induced hypopituitarism and encouraged to actively share their expertise with other physicians[15,27].

## ACKNOWLEDGMENTS

These studies have been performed under the auspices of the Italian Society of Endocrinology, on behalf of the Study Group on Physiopathology of GH Secretion. This study has been partially supported by MIUR (Ministero Istruzione Università Ricerca, Rome, Italy), Grant No. 2003069821-004.

## BIOSKETCHES

**Gianluca Aimaretti, MD**, is a researcher at the Division of Endocrinology and Metabolism, Department of Internal Medicine, University of Turin. Dr. Aimaretti's areas of scientific interest are clinical endocrinology, hypopituitarism, and growth hormone deficiency.

**Ezio Ghigo, MD**, is full professor of Endocrinology and Metabolism and Chairman of the Department of Internal Medicine of the University of Turin, Italy. Dr. Ghigo's areas of scientific interest are clinical neuroendocrinology, ghrelin, endocrinology of aging, endocrinology of obesity, and anorexia nervosa.
